# Blepharoptosis after modern phacoemulsification cataract surgery: incidence, risk factors, and underlying mechanisms

**DOI:** 10.1097/j.jcrs.0000000000001997

**Published:** 2025-06-17

**Authors:** Raphael Kilian, Andrea Taloni, Valentino De Ruvo, Rossella Spena, Massimo Busin, Cristina Bovone

**Affiliations:** From the Department of Translational Medicine and for Romagna, University of Ferrara, Ferrara, Italy (Kilian, Taloni, De Ruvo, Spena, Busin, Bovone); Moorfields Eye Hospital, London, UK (Kilian); Department of Ophthalmology, Ospedali Privati Forlì “Villa Igea”, Forlì, Italy (Kilian, Taloni, De Ruvo, Spena, Busin, Bovone); Department of Ophthalmology, Southend University Hospital, Southend-on-Sea, UK (De Ruvo).

## Abstract

**Topic:**

This was a review of the available literature on blepharoptosis after modern phacoemulsification cataract surgery with intraocular lens (IOL) implantation.

**Clinical Relevance:**

Blepharoptosis is a frequently underestimated complication of ocular surgery.

**Methods:**

This review followed PRISMA guidelines and was prospectively registered in PROSPERO (CRD420261348501). A literature search was conducted in PubMed/MEDLINE and the Cochrane Central Register of Controlled Trials, from database inception to February 2026. Studies evaluating postoperative ptosis after modern phacoemulsification with IOL implantation were included. Extracted data focused on the incidence of ptosis and associated risk factors.

**Results:**

8 studies, including a total of 863 eyes, met the inclusion criteria. 5 studies reported the exact incidence of ptosis, whereas the remaining studies only compared preoperative and postoperative marginal reflex distance 1 (MRD1). Among studies reporting incidence, rates ranged from 0% to 10.7%, with 4 of the 5 studies (674/702 eyes, 96%) reporting incidences ≤4.1%. None of the studies comparing preoperative and postoperative MRD1 measurements found a statistically significant difference. Lower preoperative levator palpebrae superioris function and higher preoperative MRD1 were identified as potential risk factors. Conversely, surgical duration and lid speculum type were not consistently associated with ptosis. Studies using exclusively topical anesthesia reported either no ptosis or no significant change in MRD1.

**Conclusions:**

Postoperative ptosis after modern cataract surgery has a low but not negligible incidence. Preoperative eyelid characteristics and the type of anesthesia may influence its occurrence. Postoperative ptosis should be routinely discussed during preoperative counselling.

## Abstract 2

Postoperative ptosis after modern cataract surgery is uncommon but clinically relevant, with risk mainly related to preoperative eyelid characteristics and anesthesia rather than surgical factors.

Postoperative blepharoptosis is a well-recognized complication of any type of anterior segment surgery. Historically, cataract extraction was associated with substantial rates of upper eyelid malposition, particularly during the era of extracapsular cataract extraction, with early reports describing postoperative ptosis in up to 44% of cases.^1^ The transition to modern clear-corneal, sutureless phacoemulsification has significantly reduced surgical manipulation and periocular tissue trauma, and consequently the reported incidence of postoperative blepharoptosis. Indeed, some prospective studies have documented an incidence as low as 0% after contemporary phacoemulsification techniques.^2^

Nevertheless, some investigations continue to report higher rates of postoperative ptosis, even in the setting of modern small-incision cataract surgery, with incidences approaching 10% in certain cohorts.^3^ The pathophysiology is presumed to be multifactorial, with proposed mechanisms including aponeurotic dehiscence of the levator palpebrae superioris (LPS), potentially resulting from prolonged mechanical compression by eyelid specula during longer surgical procedures, as well as possible myotoxic or neurotoxic effects of regional anesthetic agents.^4^

Given the increasing global volume of cataract surgery, even complications of relatively low incidence may carry significant functional, cosmetic, and medico-legal implications. The present review examines the available literature on blepharoptosis after modern phacoemulsification with intraocular lens (IOL) implantation, aiming to clarify the incidence of this complication and critically appraise the evidence regarding potential risk factors, with the goal of improving preoperative counselling and patient expectations.

## METHODS

An electronic literature search was conducted using PubMed/MEDLINE and the Cochrane Central Register of Controlled Trials, from database inception to February 2026, using combinations of the terms “ptosis,” “blepharoptosis,” “cataract surgery,” and “phacoemulsification.” This review followed PRISMA guidelines and was prospectively registered in PROSPERO (CRD420261348501) (Figure 1).^5^

**Figure 1.**
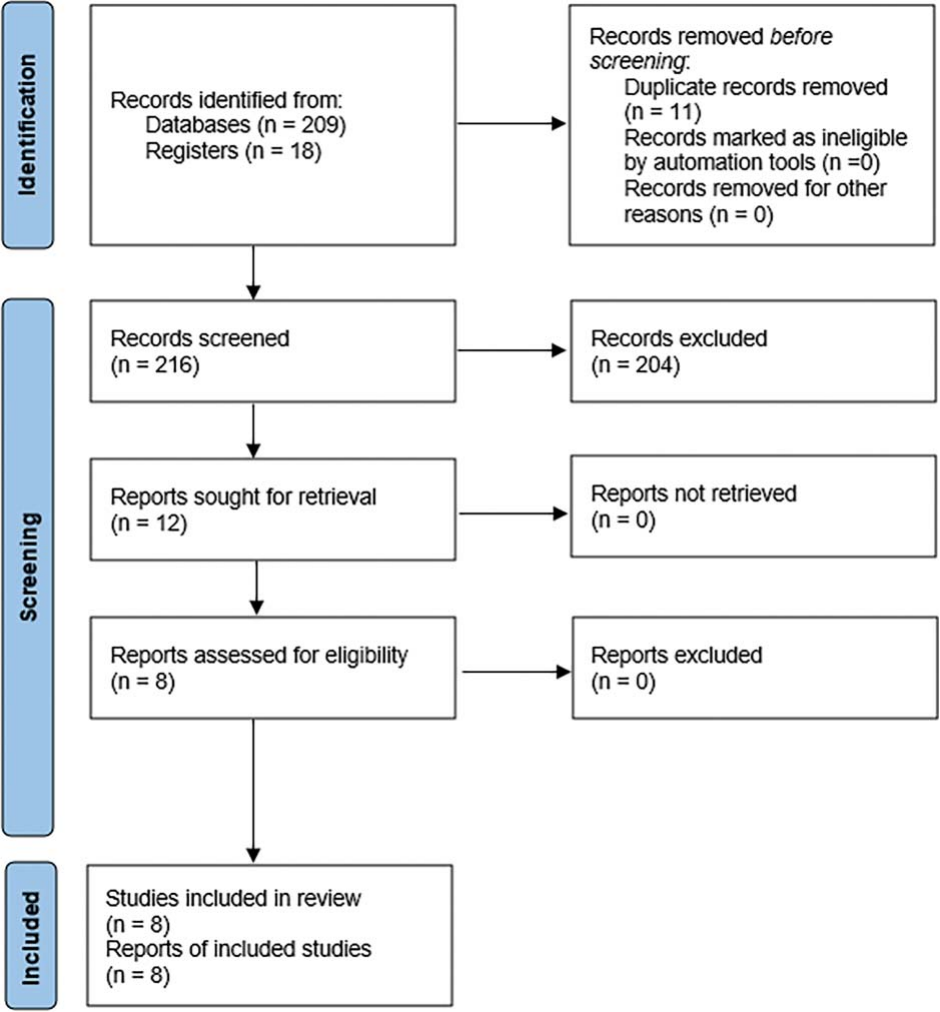
Flow diagram of study selection based on PRISMA 2020 statement.

Studies were eligible for inclusion if they were written in English, conducted in vivo on human participants, and were prospective or retrospective in design. Included studies evaluated blepharoptosis after modern phacoemulsification cataract surgery with IOL implantation. Studies exclusively analyzing extracapsular cataract extraction, intracapsular cataract extraction, manual small-incision cataract surgery, or procedures involving superior rectus bridle sutures were excluded. In studies that included mixed surgical techniques, only data corresponding to modern clear-corneal phacoemulsification with IOL implantation were extracted and analyzed separately where possible. Studies for which only an abstract was available were also excluded.

After eligibility screening, 2 independent reviewers extracted data on study characteristics, methodology, and outcomes. Any discrepancies were resolved by consensus with a third reviewer. The methodological strength of included studies was graded according to the Oxford Centre for Evidence-Based Medicine levels of evidence.^6^ Levels of evidence were 1A—systematic reviews (with homogeneity of randomized controlled trials), 1B—randomized controlled trials, 2A—systematic reviews (with homogeneity of cohort studies), 2B—individual cohort studies, 2C—outcome researches, 3A—systematic review of case–control studies, 3B—individual case–control studies, and 4—case series.

The risk of bias of included studies was assessed using the Newcastle–Ottawa Scale. This tool evaluates studies across 3 domains: selection of study groups, comparability of cohorts, and assessment of outcomes.

The primary objective of this review was to determine the reported incidence of blepharoptosis after phacoemulsification cataract surgery. Generally, clinically significant ptosis is defined as a reduction in marginal reflex distance 1 (MRD1), measured as the distance between the upper eyelid margin and the pupillary light reflex in primary gaze, of ≥2 mm compared with the preoperative measurement.^7^ Given the heterogeneity of the included studies, we reported the definition of ptosis for each study, separately.

Secondary objectives included synthesis and discussion of reported risk factors for postoperative ptosis found through univariate or multivariate analysis by each study, particularly focusing on patient-related factors (age, sex, preoperative MRD1, and levator function), surgical variables (type of speculum and duration of surgery), and type of anesthesia (topical vs local). Only statistically significant associations reported by the original authors were considered evidence of a potential risk factor. Given the heterogeneity of study designs, ptosis definitions, and follow-up intervals, quantitative pooling or meta-analysis was not performed.

## RESULTS

The electronic database search identified 227 records. After screening titles, abstracts, and full texts for eligibility, 8 studies met the inclusion criteria. These studies included a total of 863 eyes undergoing modern phacoemulsification cataract surgery with IOL implantation.

Included studies were published between 2002 and 2025 and consisted of both prospective and retrospective observational studies. Follow-up duration ranged from 6 weeks to 24 months, with most studies reporting outcomes at 3 to 6 months postoperatively. An overview of study characteristics such as anesthesia type, follow-up duration, definition of ptosis, and incidence of postoperative ptosis is presented in Table 1.

**Table 1.** Characteristics of included studies

Study	Level of evidence	Newcastle–Ottawa Scale	N	F/U (mo)	Type of anesthesia	Definition of ptosis	Incidence of ptosis at final F/U
Kashkouli et al., 2019^8^	2B	8/9	279	8.0 ± 1.9^a^	Topical: 78% Retrobulbar: 1.6% General: 20.4%	ΔMRD1 ≥2 mm	3.2%
Palanivel et al., 2024^2^	2B	7/9	238	6	Topical: 100%	ΔMRD1 or LPSF or LCD ≥2 mm/Δtwo of these ≥1 mm	0%
Marqués-Fernandez et al., 2019^9^	2B	7/9	111	6	Topical: 93% Peribulbar: 7%	ΔMRD1 ≥0.5 mm	No significant ΔMRD1
Puvanachandra et al., 2010^10^	2B	7/9	60	1.5	Topical: 100%	ΔMRD1 ≥2 mm	0%
Roddy et al., 2020^11^	2B	8/9	16	3	Retrobulbar: 100%	ΔMRD1 ≥2 mm	No significant ΔMRD1
Altieri et al., 2005^12^	4	6/9	97	24	Peribulbar: 100%	ΔMRD1 ≥2 mm	4.1%
Tamaki et al., 2016^13^	2B	8/9	34	3	Topical: 100%	ΔMRD1 ≥0.5 mm	No significant ΔMRD1
Patel et al., 2002^3^	2B	6/9	28	3^a^	Peribulbar: 100%	Unclear	10.7%

Δ = difference; LCD = lid crease distance; LPSF = levator palpebrae superioris function; MRD1 = marginal reflex distance 1

a Average

### Definition of Ptosis

There was some variability in the definition of postoperative ptosis across the included studies. Most defined clinically significant ptosis as a reduction in MRD1 of ≥2 mm compared with the preoperative measurement. This definition was used in studies by Kashkouli et al., Puvanachandra et al., Roddy et al., and Altieri et al. Others used alternative criteria. Palanivel et al. defined ptosis as a ≥2 mm change in MRD1, LPS function, or lid crease distance, or a ≥1 mm change in 2 of these parameters; whereas other studies applied more sensitive thresholds. Marqués-Fernández et al. and Tamaki et al. considered a ≥0.5 mm reduction in MRD1 as postoperative ptosis. Finally, in the study by Patel et al., the authors did not clearly specify whether a ≥2 mm change from baseline was required in 1 or all of the 3 analyzed parameters (ie, palpebral aperture, levator function, or skin crease).

### Incidence

Four of the 8 studies reported outcomes at 6 months or longer after surgery (725/863 eyes, 84%). Three studies reported outcomes at 3 months (78/863 eyes, 9%), and 1 study reported results at 6 weeks (60/863 eyes, 7%).

Five studies reported the exact incidence of postoperative ptosis, whereas the remaining studies only compared preoperative and postoperative MRD1 values (Table 1). Among the studies reporting incidence, rates ranged from 0% to 10.7%. Notably, 4 of the 5 studies (674/702 eyes, 96%) reported an incidence of ≤4.1%. By contrast, none of the studies comparing preoperative and postoperative MRD1 measurements found a statistically significant difference.

### Risk Factors

Two studies identified preoperative eyelid characteristics as predictors of postoperative ptosis. Lower preoperative levator function was associated with an increased risk of postoperative ptosis in the cohort reported by Kashkouli et al., while a higher preoperative MRD1 was associated with a greater postoperative reduction in eyelid height in the study by Tamaki et al., suggesting that patients with higher baseline eyelid position may demonstrate a larger measurable decrease after surgery.

Several intraoperative risk factors were evaluated across the included studies, such as surgical duration, surgeon experience, incision location, and use of subconjunctival steroid injection, but none was found to be consistently associated with postoperative ptosis. Surgical duration, in particular, was only evaluated as a potential risk factor by Kashkouli et al. and Marqués-Fernandez et al.; although Palanivel et al. excluded eyes with surgeries lasting longer than 10 minutes, all eyes included by Patel et al. had a surgery lasting between 30 and 40 minutes.

The type of eyelid speculum varied among studies, including Lieberman, Barraquer wire, Park, Castroviejo, and flexible EzSpec designs. However, no consistent association between speculum type and persistent postoperative ptosis was demonstrated.

Different anesthesia techniques were reported across the included studies, including topical, peribulbar, retrobulbar, and general anesthesia (Table 1). Although no statistically significant association between anesthesia type and postoperative ptosis was demonstrated, studies in which topical anesthesia was used in 100% of cases reported either a 0% incidence of ptosis or no significant difference between preoperative and postoperative MRD1 measurements. By contrast, the 2 studies in which all procedures were performed under peribulbar anesthesia reported incidences of postoperative ptosis of ≥4.1%.

## DISCUSSION

Blepharoptosis is a frequently underrecognized and underestimated complication of ocular surgery. The available literature on this topic remains relatively sparse and in many cases methodologically limited. With more than 20 million surgeries performed every year, cataract surgery is one of the most commonly performed surgical procedures worldwide, and in recent years, refractive lens exchange with implantation of premium IOLs has gained popularity.^14,15^ In this context, even complications that may seem primarily cosmetic or only mildly functional, such as postoperative ptosis, can have a significant impact on overall patient satisfaction and perceived surgical success.

This review highlights some heterogeneity among studies, particularly regarding the definition of postoperative ptosis. Nevertheless, the available evidence suggests that the incidence of clinically significant chronic postoperative ptosis generally ranges between 0% and 4%, and the occurrence of this complication seems to be influenced primarily by preoperative LPS function and the baseline MRD1.

The pathophysiology of postoperative ptosis after cataract surgery has long been debated and remains incompletely understood. Traditionally, postoperative ptosis was associated with the use of a superior rectus bridle suture, which was used to infraduct the globe during surgery.^16^ Traction on the superior rectus may disrupt the fascial connections between the superior rectus and the LPS, potentially leading to levator aponeurosis dehiscence and eyelid drooping. However, ptosis continues to occur even in contemporary phacoemulsification techniques where bridle sutures are omitted, suggesting that additional mechanisms are involved.

In modern cataract surgery, the eyelid speculum is considered a major contributing factor.^17^ Prolonged use of a rigid speculum can exert compressive and stretching forces on the upper eyelid, resulting in mechanical trauma to the levator complex. Several mechanisms have been proposed: Compression of the eyelid against the orbital rim may impair blood flow to the levator muscle, sustained horizontal stretching of the eyelid may increase laxity, and resistance of the orbicularis oculi against the speculum may lead to levator aponeurosis dehiscence. These processes can cause mild-to-moderate ptosis with relatively preserved levator function, a pattern similar to involutional aponeurotic ptosis.

Additional contributing factors may include anesthetic-related effects, particularly with peribulbar or retrobulbar blocks.^18^ Local anesthetics can cause temporary neuromuscular blockade of the levator palpebrae superioris or myotoxic injury to muscle fibers, resulting in transient ptosis that typically resolves within weeks as muscle fibers regenerate. Similarly, postoperative eyelid edema, hematoma formation, and inflammation may contribute to early transient eyelid malposition in the immediate postoperative period, although these mechanisms are unlikely to account for persistent ptosis observed several months after surgery.^19^ In accordance with this theory, the results of this review seem to suggest that eyes undergoing topical anesthesia, although not immune, may have a lower risk of ptosis development. Still, in the past, there have been reports showing high ptosis rates in eyes undergoing anterior segment procedures under topical anesthesia only. In their series, Linberg et al. showed a 10% incidence of ptosis in a young cohort undergoing radial keratotomies under topical anesthesia.^20^ These data show that postoperative ptosis can occur even in young patients undergoing topical anesthesia, supporting a mechanical mechanism such as speculum-related trauma.

Lower preoperative levator function was associated with an increased risk of persistent postoperative ptosis in the cohort reported by Kashkouli et al. Although reduced levator function may appear unexpected given that postoperative ptosis is classically considered an aponeurotic/dehiscence process, subclinical age-related levator changes or a more vulnerable levator–aponeurotic complex may predispose certain patients to clinically evident postoperative ptosis after surgical manipulation.

Given the lack of studies systematically analyzing at the same time risk factors for ptosis after modern phacoemulsification and IOL implantation, such as length of surgery, type of speculum, and type of anesthesia, further prospective research including possible randomized controlled trials, in this field is required.

Furthermore, it should also be noted that some included studies defined postoperative ptosis as a reduction in MRD1 of only ≥0.5 mm. Although this approach increases sensitivity for detecting subtle postoperative eyelid changes, such small differences may fall within the range of normal interexamination variability and may not be clinically perceptible to patients. By contrast, studies using a ≥2 mm threshold are likely to better reflect clinically significant ptosis with functional or aesthetic relevance. Therefore, caution is warranted when comparing incidence rates across studies using different diagnostic criteria.

In addition, variability in postoperative follow-up duration and differences in MRD1 assessment methods across studies may have influenced the reported results and represent limitations of this study.

To conclude, postoperative ptosis should be mentioned among the possible postoperative complications during preoperative assessment and consenting, especially in younger patients undergoing phacoemulsification for refractive purposes, where the aesthetic result might play a more significant role. In addition, results of this review show that from a medico-legal standpoint, awareness and documentation of preoperative eyelid position may be prudent because this seems to influence the incidence of postoperative ptosis. More studies are necessary to further investigate the risks factors for this condition.

## References

[1] Singh SK Sekhar GC Gupta S. Etiology of ptosis after cataract surgery. J Cataract Refract Surg. 1997;23(9):1409–1413. doi:10.1016/s0886-3350(97)80124-1 9423917

[2] Palanivel S Jayagayathri R Yadalla D. The impact of speculum uses on the incidence of blepharoptosis in patients undergoing phacoemulsification surgery: a prospective analysis. Indian J Ophthalmol. 2025;73(2):210–213. doi:10.4103/IJO.IJO_1476_24 PMC11991550 39853141

[3] Patel JI Blount M Jones C. Surgical blepharoptosis: the bridle suture factor? Eye (Lond). 2002;16(5):535–537. doi:10.1038/sj.eye.6700022 12194064

[4] Mehat MS Sood V Madge S. Blepharoptosis following anterior segment surgery: a new theory for an old problem. Orbit. 2012;31(4):274–278. doi:10.3109/01676830.2012.681750 22642903

[5] Page MJ McKenzie JE Bossuyt PM Boutron I Hoffmann TC Mulrow CD Shamseer L Tetzlaff JM Akl EA Brennan SE Chou R Glanville J Grimshaw JM Hróbjartsson A Lalu MM Li T Loder EW Mayo-Wilson E McDonald S McGuinness LA Stewart LA Thomas J Tricco AC Welch VA Whiting P Moher D. The PRISMA 2020 statement: an updated guideline for reporting systematic reviews. BMJ. 2021;372:n71. doi:10.1136/bmj.n71 PMC8005924 33782057

[6] Oxford Centre for Evidence-Based Medicine. Oxford Centre for Evidence-Based Medicine levels of evidence. 2009. Accessed March 1, 2026. http://www.cebm.net/oxford-centre-evidence-based-mdicine-levelsevidence-march-2009/

[7] Paris GL Quickert MH. Disinsertion of the aponeurosis of the levator palpebrae superioris muscle after cataract extraction. Am J Ophthalmol. 1976;81(3):337–340. doi:10.1016/0002-9394(76)90250-6 1258958

[8] Kashkouli MB Abdolalizadeh P Es'haghi A Nilforushan N Aghaei H Karimi N. Postoperative blepharoptosis after modern phacoemulsification procedure. Am J Ophthalmol. 2020;213:17–23. doi:10.1016/j.ajo.2019.12.023 31926884

[9] Marqués-Fernández V Garcia-Zamora M Calabuig-Goena M Diez-Montero C Schellini SA Khandekar R Galindo-Ferreiro A. An objective evaluation of the upper eyelid position after phacoemulsification cataract surgery. Semin Ophthalmol. 2019;34(6):442–445. doi:10.1080/08820538.2019.1643379 31314625

[10] Puvanachandra N Hustler A Seah LL Tyers AG. The incidence of ptosis following extracapsular and phacoemulsification surgery: comparison of two prospective studies and review of the literature. Orbit. 2010;29(6):321–323. doi:10.3109/01676830.2010.522297 21158573

[11] Roddy GW Zhao B Wang F Fang C Khanna SS Bajric J Khanna CL. Increased rate of ptosis following glaucoma drainage device placement and other anterior segment surgery: a prospective analysis. Graefes Arch Clin Exp Ophthalmol. 2020;258(7):1533–1541. doi:10.1007/s00417-020-04630-x 32103333

[12] Altieri M Truscott E Kingston AE Bertagno R Altieri G. Ptosis secondary to anterior segment surgery and its repair in a two-year follow-up study. Ophthalmologica. 2005;219(3):129–135. doi:10.1159/000085244 15947497

[13] Tamaki R Gosho M Mizumoto K Kato N Zako M. Influence of upper and temporal transconjunctival sclerocorneal incision on marginal reflex distance after cataract surgery. BMC Ophthalmol. 2016;16:95. doi:10.1186/s12886-016-0286-1 PMC4937608 27387207

[14] Han X Zhang J Liu Z Tan X Jin G He M Luo L Liu Y. Real-world visual outcomes of cataract surgery based on population-based studies: a systematic review. Br J Ophthalmol. 2023;107(8):1056–1065. doi:10.1136/bjophthalmol-2021-320997 PMC10359559 35410876

[15] Stern B Gatinel D. Presbyopia correction in lens replacement surgery: a review. Clin Exp Ophthalmol. 2025;53(6):668–681. doi:10.1111/ceo.14535 PMC12326228 40295166

[16] Loeffler M Solomon LD Renaud M. Postcataract extraction ptosis: effect of the bridle suture. J Cataract Refract Surg. 1990;16(4):501–504. doi:10.1016/s0886-3350(13)80807-3 2199666

[17] Crosby NJ Shepherd D Murray A. Mechanical testing of lid speculae and relationship to postoperative ptosis. Eye (Lond). 2013;27(9):1098–1101. doi:10.1038/eye.2013.133 PMC3772367 23788211

[18] Hosal BM Tekeli O Gürsel E. Eyelid malpositions after cataract surgery. Eur J Ophthalmol. 1998;8(1):12–15. doi:10.1177/112067219800800104 9590589

[19] Ahuero AE Hatton MP. Eyelid malposition after cataract and refractive surgery. Int Ophthalmol Clin. 2010;50(1):25–36. doi:10.1097/IIO.0b013e3181c55150 20057293

[20] Linberg JV McDonald MB Safir A Googe JM. Ptosis following radial keratotomy performed using a rigid eyelid speculum. Ophthalmology. 1986;93(12):1509–1512. doi:10.1016/s0161-6420(86)33529-2 3808612

